# Twitter-Delivered Behavioral Weight-Loss Interventions: A Pilot Series

**DOI:** 10.2196/resprot.4864

**Published:** 2015-10-23

**Authors:** Sherry L Pagoto, Molly E Waring, Kristin L Schneider, Jessica L Oleski, Effie Olendzki, Rashelle B Hayes, Bradley M Appelhans, Matthew C Whited, Andrew M Busch, Stephenie C Lemon

**Affiliations:** ^1^ Division of Preventive and Behavioral Medicine Department of Medicine University of Massachusetts Medical School Worcester, MA United States; ^2^ Departments of Quantitative Health Sciences and Obstetrics & Gynecology University of Massachusetts Medical School Worcester, MA United States; ^3^ Department of Psychology College of Health Professions Rosalind Franklin University of Medicine & Science North Chicago, IL United States; ^4^ Department of Preventive Medicine Rush University Medical Center Chicago, IL United States; ^5^ Department of Psychology East Carolina University Greenville, NC United States; ^6^ The Miriam Hospital Centers for Behavioral and Preventive Medicine; Department of Psychiatry and Human Behavior Alpert Medical School of Brown University Providence, RI United States

**Keywords:** social networks, Twitter, obesity, weight loss, online social networking, peer-to-peer health care, digital health

## Abstract

**Background:**

Lifestyle interventions are efficacious at reducing risk for diabetes and cardiovascular disease but have not had a significant public health impact given high cost and patient and provider burden.

**Objective:**

Online social networks may reduce the burden of lifestyle interventions to the extent that they displace in-person visits and may enhance opportunities for social support for weight loss.

**Methods:**

We conducted an iterative series of pilot studies to evaluate the feasibility and acceptability of using online social networks to deliver a lifestyle intervention.

**Results:**

In Study 1 (n=10), obese participants with depression received lifestyle counseling via 12 weekly group visits and a private group formed using the online social network, Twitter. Mean weight loss was 2.3 pounds (SD 7.7; range -19.2 to 8.2) or 1.2% (SD 3.6) of baseline weight. A total of 67% (6/9) of participants completing exit interviews found the support of the Twitter group at least somewhat useful. In Study 2 (n=11), participants were not depressed and were required to be regular users of social media. Participants lost, on average, 5.6 pounds (SD 6.3; range -15 to 0) or 3.0% (SD 3.4) of baseline weight, and 100% (9/9) completing exit interviews found the support of the Twitter group at least somewhat useful. To explore the feasibility of eliminating in-person visits, in Study 3 (n=12), we delivered a 12-week lifestyle intervention almost entirely via Twitter by limiting the number of group visits to one, while using the same inclusion criteria as that used in Study 2. Participants lost, on average, 5.4 pounds (SD 6.4; range -14.2 to 3.9) or 3.0% (SD 3.1) of baseline weight, and 90% (9/10) completing exit interviews found the support of the Twitter group at least somewhat useful. Findings revealed that a private Twitter weight-loss group was both feasible and acceptable for many patients, particularly among regular users of social media.

**Conclusions:**

Future research should evaluate the efficacy and cost-effectiveness of online social network-delivered lifestyle interventions relative to traditional modalities.

## Introduction

Lifestyle interventions (ie, behavioral weight-loss interventions) have had established efficacy for over a decade, but are still not widely disseminated largely due to high cost and patient and provider burden [1-3]. Online social networks provide an alternative mode for delivery of lifestyle counseling which may reduce patient visits, the main source of cost and burden of traditional modalities. Interactions in online social networks are frequent, brief, and asynchronous because users log into their online communities during downtime or when they simply feel a need for social connection. As such, social media has become embedded into many people’s daily lives. Online social networks may then provide a means to embed health behavior-change programming into people’s daily lives [4]. Another advantage of using social media to deliver lifestyle interventions is that it increases opportunities for patients to receive social support for their weight-loss efforts [5], which may be particularly important for socially isolated populations, such as those with depression [6].

Most studies have used an online social network as an adjunct to traditionally delivered weight-loss programs, either by conducting scheduled group chats online [7,8] or by providing a message board/forum for participants to submit questions and chat [9,10]. One study used an online social network as an adjunct to a podcast-delivered intervention but did not find that it improved outcomes relative to a podcast-only condition [11], while two ongoing studies are using an online social network as the main intervention delivery modality for weight-loss interventions [12,13]. Two recent systematic reviews of 12 and 20 studies, respectively, revealed that the impact of the online social network component of a weight-loss intervention has never been isolated [14,15]. Three studies showed that engagement in the online social network predicted greater weight loss, which suggests that the social network component may have a role in promoting better outcomes [16-18]. Although research on the role of online social networks in facilitating weight loss shows promise, further studies are required to determine the ideal way to utilize the social network (ie, as an adjunct versus the sole treatment modality), participants most likely to prefer this modality, and who will benefit the most.

Another gap in the literature is that few social network weight-loss studies have used mainstream social networking platforms like Facebook or Twitter [14,15]. Investigator-designed websites may lack the technological sophistication and usability of mainstream platforms, which have undergone many years of refinement by expert developers [14]. Leveraging widely used and freely available social networking platforms in health behavior interventions can increase the sustainability and dissemination potential of interventions, and eliminates the costs associated with developing new platforms that can quickly become obsolete.

**Figure 1:**
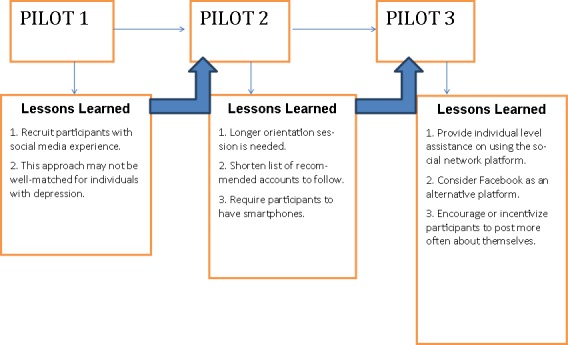
Flow of research.

This report describes an iterative series of three pilot studies in which we evaluated the feasibility and acceptability (eg, engagement and retention) of using Twitter, a mainstream commercial online social network, as an adjunct to a traditional group visit-delivered lifestyle intervention in depressed and nondepressed samples, and as the primary intervention modality in a nondepressed sample. This pilot series provided multiple opportunities to iteratively refine the intervention based on our experiences in each study. We present the methodology, results, and lessons learned for each study in the order they were performed to demonstrate how this line of work evolved (see Figure 1). The overarching goal of this work was to determine for whom online social networks might be most acceptable and how much of the intervention is feasible to deliver via the online social network. In each pilot, we created a private social community on Twitter as either an adjunctive component of an in-person lifestyle intervention or as the primary intervention modality. Focus groups were conducted after each pilot to get feedback on what participants liked and disliked about the social network aspect of the program. Survey items explored the acceptability of the social network and of various forms of engagement. Finally, to characterize the social influence of Twitter groups relative to other relationships, we compared participants’ perceptions of social support and negativity regarding weight from their Twitter group against perceived social support and negativity in preexisting in-person relationships (ie, family and friends).

## Methods

### Overview of Pilot Series

In Study 1, we enrolled adults with obesity and depression. Individuals with depression tend to have greater difficulty losing weight [19] and tend to feel more socially isolated [20]. A supportive social network could provide participants with social experiences devoid of weight stigma, which can increase depressive symptoms and inhibit weight loss [21,22]. The intervention involved 12 weekly visits and an adjunctive online social network component (via Twitter) that connected participants and counselors. In between group visits, counselors used Twitter to continue conversations about the topic of the week, share links to relevant content, provide support when participants reported progress or struggles, and interact with participants who missed visits. We hypothesized that individuals with depression would find the online social network an acceptable and desirable means of eliciting extra support in between group visits. Given the challenges reported by the sample in Study 1 and an aim to further increase generalizability, in Study 2 we enrolled adults who were not depressed and who had regular social media experience into the same intervention. Given the high level of acceptability of the online social network in this population, in Study 3 we enrolled participants with similar characteristics as those in Study 2 into a lifestyle intervention that was delivered almost exclusively via the online social network. Focus groups and surveys following each intervention evaluated acceptability and participants' likes and dislikes about the program. The University of Massachusetts (UMass) Medical School Human Subject Committee approved all study procedures.

### Study 1 Methods

#### Recruitment and Screening

In August 2012, participants were recruited though the local community and the UMass Medical School. Study flyers were posted on billboards throughout the community and university. Online advertisements were posted on local advertisement sites and the university distribution list. The study advertisement was also distributed via recruitment-specific newsletters sent to the community. Individuals responding to ads were screened by phone for eligibility. Eligible and interested individuals were scheduled for a baseline visit lasting 90 minutes during which they provided written informed consent and their height and weight were taken. Participants also completed surveys including the following: demographics, Beck Depression Inventory II (BDI-II) [23], medical history, medications, and social support for weight loss (eg, Weight Management Support Inventory [WMSI]) [24].

#### Inclusion and Exclusion Criteria

Participants were required to have a BDI-II score of >13 (mild or greater depression), be 18 to 65 years of age, have a body mass index (BMI) of 30 to 45 kg/m^2^, have written clearance from their primary care provider to participate, and have Internet access in their home. Participants were excluded if they had initiated an antidepressant medication within past 2 months, had plans to move during the study, were pregnant or lactating, had severe mental illness (eg, bipolar disorder or schizophrenia) or a BDI-II score >30, had bariatric surgery, had a condition that precludes lifestyle changes, were taking a medication affecting weight, had type 1 or type 2 diabetes, or were active Twitter users (ie, tweeted in the last 3 months). Recruiting both users and nonusers would have essentially created two subgroups that, with a small sample, might not adequately capture the experience of either, and recruiting only active Twitter users would have drastically limited the sample since only 23% of adults use Twitter [25]. Additionally, Twitter is generally used as a public forum, thus active users would not be able to use their current accounts but instead would have to start a second account to participate in the study since participation required the use of privacy settings. To reduce this variability in the small sample, we recruited people not actively using Twitter.

#### Intervention

Participants were enrolled into a 12-week weight-loss intervention involving an orientation visit, 12 weekly group counseling visits, and access to a private online Twitter group. The orientation visit lasted 90 minutes and assisted participants with setting up a Twitter account and learning how to use the mobile app, MyFitnessPal [26], to track dietary intake and physical activity. Participants developed their Twitter profiles using avatars and pseudonyms and posted their first tweets. They were encouraged to (1) tweet questions to the group and group leaders, (2) participate in discussions (eg, “What is the biggest challenge you have with holiday eating?”) and challenges (eg, “Post a pic you took while exercising outside!”) put forth by group leaders, (3) report their exercise, and (4) share their victories and challenges. Participants were encouraged to follow a list of 50 other weight- and health-related Twitter accounts and were provided a list of such accounts that were vetted by the investigators to insure the legitimacy of information provided.

#### Group Visits

A shortened version of the Diabetes Prevention Program (DPP) Lifestyle Intervention [27] was delivered by a clinical psychologist and exercise physiologist over the course of 12 weekly, 90-minute group counseling visits. Participants were given a calorie goal based on their basal metabolic rate that was adjusted to create an approximately 1- to 2-pound weight loss per week. They were encouraged to increase their exercise gradually to 150 minutes per week of moderate-intensity physical activity. Each participant was weighed privately at the beginning of group visits.

#### Online Group

The group leaders posted discussion topics each day based on the topic of discussion in the group visits. They also posted relevant articles, healthy recipes, information about healthy community events (eg, 5K races), and launched challenges and quizzes. Group leaders also directly tweeted participants who had not engaged in the social network in 1 week by mentioning their names in tweets (eg, “@puppymama, how are you doing?”) in order to draw the participants back into the conversation.

#### Measures

##### Weight

Weight was assessed using a digital scale (Scale-Tronix, Model 5002, White Plains, NY) with participants wearing light clothing.

##### Retention

A dropout was defined as an individual who exited the study before it ended, meaning they discontinued both group visits and posting tweets in the online group, and they failed to attend a follow-up assessment visit.

##### Online Social Network Engagement

Engagement was defined broadly as the average number of Twitter posts and/or replies made by a participant during the 12-week program.

##### Acceptability

Survey items were developed to evaluate how helpful the Twitter social network was as a source of support and information, how likely they would be to continue using Twitter after the study, and how much they liked various forms of engagement, including posting updates about themselves, asking questions, replying to others' questions, and reading others' posts. Responses were on a scale from 0 (not at all/not at all likely) to 10 (very much/very likely).

##### Weight-Loss Social Support

Participants were asked to rate weight-loss social support they experienced from three relationship categories: Twitter friends (ie, group members and anyone else they may have decided to follow), in-person friends, and family [28]. The following definition of in-person friends was given: “Any friends that you interact with in person, meaning you see them and spend time with them.” Three of the four subscales of the Weight Management Support Inventory, a validated weight-loss social support scale, were administered [24]: the informational (seven items), emotional (six items), and appraisal (three items) subscales. While these three subscales have been shown to be valid in online weight-loss programs, the instrumental support subscale was not, so we did not include it [29]. We eliminated three items from the original appraisal scale because, as worded, they refer to physical observations of an individual (eg, “others tell me I look like I’m in better shape”), which is not possible in an online social network. Each subscale included both a frequency (ie, how often did this occur in in the past 4 weeks) and a helpfulness dimension (ie, how helpful was each). Frequency responses were on a 5-point Likert scale from *not at all* to *about every day*. The helpfulness responses were on a 5-point Likert scale from *not at all helpful* to *extremely helpful*.

We also asked questions regarding social support for weight loss and negative social influence using items found to be internally consistent in our previous work [28]. These questions used 5-point Likert scales with responses ranging from *very much disagree* to *very much agree*. Single-item questions were used to assess five different aspects of positive social support—comfort, helpfulness, support, information, and fun—in regard to people in each of the three relationship categories. As in previous research [28], items were summed to create a total score with higher scores reflecting more positive social influence. Two items were used to assess negative social influence, one that asked participants to rate how embarrassed they feel discussing weight-related issues with people in each relationship category, and another that asked participants to rate how judgmental they feel people in each relationship category are when it comes to weight-related issues. These items were not combined into a composite because they did not have high internal consistency.

#### Focus Group

#### Analytic Plan

All outcomes were summarized with descriptive statistics. Two investigators (JO, SP) reviewed focus group responses and developed themes for each category of responses. Responses were then coded by two investigators (JO, RH) and discrepancies were discussed until consensus was reached. Quantitative analyses were conducted using SPSS version 22.0 (IBM Corp, Armonk, NY). Qualitative analyses were conducted using NVivo version 10.0 (QSR International, Doncaster, Australia).

A 1-Hour focus group led by an experienced focus group facilitator was convened after the 12-week follow-up visit to ask participants what they liked best and least about the online aspect of the program and their suggestions for improvement. A note taker was present and the focus group was recorded and later transcribed for the coders. Participants were compensated for attendance.

### Study 1 Results

#### Overview

The sample (n=10) was 90% female (9/10), had a mean age of 46.2 years (SD 10.9), and was 80% (8/10) non-Hispanic white with a mean BMI of 35.86 kg/m^2^ (SD 4.62) and a mean BDI-II score of 16.82 (SD 11.90) (see Table 1). Most participants (8/10, 80%) were employed full time. A total of 30% (3/10) of participants had no social media accounts, 40% (4/10) had one social media account, and 30% (3/10) had more than one social media account. Among those who used social media, 70% (7/10) reported logging into their accounts at least once per day.

#### Measures

##### Retention, Attendance, and Engagement

One participant dropped out, resulting in a 90% (9/10) retention rate. On average, participants attended 7.8 of the 10 group visits (SD 2.2; range 3 to 10). Participants posted an average of 110.7 tweets (SD 112.4; median 66.6, interquartile range [IQR] 167) over the course of 12 weeks.

##### Weight Loss

Participants lost a mean of 2.3 pounds (SD 7.7; range -19.2 to 8.2) or 1.2% (SD 3.6) of their baseline weight. A total of 2 out of 10 (20%) participants lost clinically significant weight (ie, ≥5% of baseline weight), 40% (4/10) were at the same weight as they were at baseline, and 20% (2/10) gained 1 pound or more (see Table 2).

##### Acceptability

Of the 9 participants that completed the acceptability survey, two-thirds (6/9, 67%) reported that they found the Twitter group to be at least a somewhat useful source of support and information and that they are at least somewhat likely to continue to use Twitter after the study has ended (see Table 3). Just over half (5/9, 56%) reported to at least somewhat like posting a status update about themselves or posing a question to the group. Two-thirds (6/9, 67%) reported to at least somewhat like replying to other people’s questions and just over half (5/9, 56%) at least somewhat liked seeing other people’s posts.

##### Weight-Loss Social Support

At follow-up, participants' scores on the WMSI emotional subscales for frequency and helpfulness were not significantly different by relationship category (*P*=.12 to .90), however informational support subscales for frequency and helpfulness were significantly higher for the Twitter group relative to family (*P*=.01 and .02, respectively) and friends (*P*=.02 and .01, respectively). Appraisal support scales for frequency and helpfulness were not significantly different by relationship category (*P*=.05 to .73). Participant ratings of weight-related positive social influence (*P*=.10) and negative social influence, including embarrassment (*P*=.08), and how judgmental the people are (*P*=.06) did not differ by relationship category (ie, Twitter group, family, and friends) (see Table 4).

#### Focus Group

A total of 7 out of 10 participants (70%) attended the focus group. Several themes emerged from a total of 16 responses about what participants liked most about the online aspect of the program. Major themes were each endorsed by 3 of the 7 (43%) participants and included the following: being in a group of people with shared interests and goals (eg, “I liked that we all had a common goal.”), enjoying interacting with others (eg, “I like the social interaction and openness of the group.”), and social support for weight loss (eg, “Support from the group was uplifting.”). Minor themes, each endorsed by 2 out of 7 participants (29%), included the responsiveness of others when they posted (eg, “I liked the fast and immediate responses I got.”), anonymity (eg, “No one knows who you are.”), and the information they received (eg, “Lots of tips, good information, and recipes.”). Finally, 1 participant out of 7 (14%) said they did not like anything about the online piece (eg, “I don’t tweet.”).

Several themes emerged from a total of 11 responses about what participants liked least about the online aspect of the program. The major theme, endorsed by 3 out of 7 participants (43%), was that there was not anything they disliked about the program. Three minor themes emerged, each endorsed by 2 of 7 participants (29%), and included difficulty understanding how to use Twitter, finding the feed to be overwhelming, and having limited access to Twitter due to work or lack of mobile phone. Finally, 1 participant of 7 (14%) mentioned that she did not feel comfortable posting about herself.

#### Lessons Learned From Pilot 1

Because participants in Study 1 lost very little weight on average and reported many barriers to using the online social network, we concluded that perhaps the extra effort of this aspect of the intervention was not adding value and even possibly detracting value. Previous research shows that individuals with depression have higher rates of treatment failure [19] and this study seemed to be no exception. Another factor affecting the perceived effort of using the online social network was the lack of social media experience in this sample. Based on these results, for Study 2 we decided to keep the intervention the same but to enroll adults who did not have depression and who had a higher degree of social media experience as indicated by their being regular Facebook users. Facebook is the most highly used online social network [25], thus this inclusion criteria would not sufficiently hinder recruitment and addressed concerns we had regarding enrolling existing Twitter users who would then need to create a second private account.

### Study 2 Methods

#### Recruitment and Screening

In April 2013, the same recruitment and screening procedures were used as in Study 1, except the BDI-II cutoff was not used.

#### Inclusion and Exclusion Criteria

Eligibility criteria included active Facebook and mobile phone users aged 18 to 65 years who had a BMI between 30 and 45 kg/m^2^ and approval from their primary care physician to participate. Participants were excluded if they had plans on moving during the study period, were currently pregnant or lactating, had plans to have bariatric surgery during the study period, had medical conditions preventing dietary changes or an increase in physical activity, had type 1 or type 2 diabetes, and were taking medications associated with weight gain.

#### Measures

The measures used in Study 2 were the same as those used in Study 1.

#### Intervention

Group visits and the Twitter component were carried out in the same fashion as in Study 1.

#### Analytic Plan

The analytic plan was the same as that in Study 1.

### Study 2 Results

#### Overview

Participants (n=11) were all female (100%) and largely Caucasian (9/11, 82%) with a mean age of 48.3 years (SD 12.4) and a mean BMI of 33.8 kg/m^2^ (SD 3.7). Most (10/11, 91%) were employed full time. The mean BDI-II score was 5.4 (SD 3.9), which fell well within the minimal depression range (0 to 13). The majority of participants (10/11, 91%) had more than one social media account and 64% (7/11) of social media users reported at least daily log-ins (see Table 1).

#### Measures

##### Retention, Attendance, and Engagement

Two participants dropped out, resulting in an 82% (9/11) retention rate. On average, participants attended 8.09 of the 12 group visits (SD 3.60; range 1 to 12). Participants posted an average of 121.9 tweets (SD 127.0; median 73.0, IQR 191) over the course of 12 weeks.

##### Weight Loss

Participants lost a mean of 5.6 pounds (SD 6.3; range -15 to 0) or 3.0% of their baseline weight (SD 3.4) (see Table 2). A total of 36% (4/11) of participants lost clinically significant weight (ie, ≥5% of baseline weight), 27% (3/11) lost less than 5% of their baseline weight, and 36% (4/11) had no weight change. No participants gained weight.

##### Acceptability

Of the 9 participants who completed the acceptability survey, all (100%) found the Twitter group to be at least a somewhat useful source of support and the vast majority (8/9, 89%) found it to be at least a somewhat useful source of information (see Table 3). All participants said they would be at least somewhat likely to continue with Twitter following the intervention. The vast majority of participants at least somewhat liked posting a status update (7/9, 78%), posing a question (8/9, 89%), replying to others' questions (9/9, 100%), and reading other people’s posts (9/9, 100%).

##### Weight-Related Social Support

At follow-up, emotional support subscales for frequency were significantly higher for the Twitter group relative to family (*P*=.01) and friends (*P*<.001). The emotional support subscales for helpfulness were not different between the Twitter group and family (*P*=.12), but were higher for the Twitter group than for in-person friends (*P*=.04). The informational support subscales for frequency and helpfulness were greater for the Twitter group than for both family (*P*<.001 and *P*=.01, respectively) and friends (*P*<.001 and *P*=.01, respectively). The appraisal support subscales for frequency and helpfulness did not differ by relationship category (*P*=.48 to .85). Positive social influence differed by relationship category (*P*=.005) (see Table 4). Participants rated their Twitter group as a greater source of positive social influence for weight loss compared to their family (*P*=.007) and in-person friends (*P*=.03). In terms of how embarrassed they felt discussing weight-related topics, no differences were found in participants’ ratings across relationship types (*P*=.33). Differences emerged in ratings of how judgmental they rated each relationship category (*P*=.03), such that participants rated their family as more judgmental than their Twitter group (*P*=.003).

#### Focus Group

The focus group was attended by 9 of 11 participants (82%). Several themes emerged from a total of 22 responses about what participants liked most about the online aspect of the program. Five major themes emerged. The most frequently endorsed theme, endorsed by 7 of the 9 participants (78%), was social support received from the group (eg, “For me, I need positive feedback. People would say positive, nonjudgmental things.”). The next major theme, endorsed by 5 of 9 participants (56%), was feeling nudged or inspired by others' posts (eg, “You see someone post they walked 3 miles and think, hey she’s making me look bad! I can get off my butt and do that too!”). Liking that everyone was on the same path (eg, “It’s nice to have people that I can talk to even though I don’t spend time with them I feel like I know them, we all have the same goal.”) was a major theme endorsed by 4 of 9 (44%) participants. Information (eg, “I liked getting information and recipes right in front of me.”) and instant feedback (eg, “Every time I posted a walk I would get a favorite or reply the same day and that felt good.”) were major themes, each endorsed by 3 of the 9 (33%) participants.

Two major themes emerged from a total of 15 responses about what participants liked least about the online aspect of the program. The most frequently occurring theme, endorsed by 8 of the 9 participants (89%), was the Twitter feed being overwhelming with too many tweets from too many people they were following. The other major theme was the usability of Twitter (eg, “The app had different functions than on the computer.”), endorsed by 4 of the 9 (44%) participants. A total of 3 of the 9 participants (33%) made unique comments that did not fit into the major themes. Out of the 9 participants, 1 (11%) mentioned feeling uncomfortable when strangers would send follow requests (eg, “I had this experience with some guy that started following me and I have no idea how he found me or why he would care. That bothered me.”). Out of the 9 participants, 1 (11%) said she did not know what to post (eg, “I didn’t think I really had anything to say.”) and 1 (11%) said there was not anything she disliked.

#### Lessons Learned From Study 2

Given the more promising weight losses and the higher level of acceptability in Study 2 relative to Study 1, we concluded that adults who have experience with social media and no major mental health barriers may be a promising group to attempt a more aggressive social media-delivered approach. Thus, for Study 3 we decided to deliver a far greater amount of intervention content through the online social network and to limit in-person group visits to one. We required participants to have mobile phones and experience using mobile apps to insure competence in accessing apps from mobile phones and to maximize accessibility to the group. We also extended the orientation by 30 minutes and spent more time on Twitter functions. Finally, we decided against giving participants a list of 50 accounts to follow since they seemed to find the feed too overwhelming, but rather suggested just 10 and said following the accounts was optional.

### Study 3 Methods

#### Recruitment

In October 2013, recruitment methods described for Study 1 were used.

#### Inclusion and Exclusion Criteria

Eligibility criteria included having a Facebook account, being aged 18 to 65 years, and having a BMI between 30 and 45 kg/m^2^. Participants were excluded if they had used Twitter in past 3 months, had plans to move during the study, did not have a scale at home to weigh themselves, were currently pregnant or lactating, had plans to have bariatric surgery during the study period, had medical conditions preventing dietary changes or an increase in physical activity, had type 1 or type 2 diabetes, were taking exclusionary medications, were not a current mobile phone user, or had never used a mobile app.

#### Intervention

##### Orientation

Participants were enrolled into a 12-week weight-loss intervention involving an orientation visit, a midintervention visit, and a private online group formed using Twitter. The orientation visit lasted 2 hours and was similar to the one in Studies 1 and 2 except it involved additional practice at each step because our focus group results from Studies 1 and 2 suggested that participants wanted more instruction on how to use Twitter. Participants also received their calorie, physical activity, and weight-loss goals at the orientation session.

##### Online Group

Diabetes Prevention Program Lifestyle Intervention materials were converted into online articles and links were tweeted with a byline that described the content (eg, “8 tips for healthier eating out! Which will you try this week?”). Each day, group leaders posted a new topic with accompanying links and discussions. Counselors logged in twice a day to start discussions, answer questions, provide positive reinforcement, and share content. Participants were emailed each week to elicit their self-reported weights. As in Study 1, group leaders made daily posts of relevant articles, healthy recipes, information about healthy community events (eg, 5K races), and launched challenges and quizzes. Group leaders tweeted at specific participants who had not engaged in the social network in 1 week to draw the participant back into the conversation as in Studies 1 and 2. Participants were given a list of 10 suggested Twitter accounts to follow that included healthy recipe websites (eg, @cookinglight) or popular weight-loss bloggers, but were informed this was optional.

##### Midintervention Visit

At week 6, participants attended a group visit in which group leaders did “check-ins” and problem solving with participants regarding any challenges they were experiencing in their weight-loss journey or with participating in the Twitter group.

#### Measures

The measures used in Study 3 were the same as those used in Studies 1 and 2.

#### Sample

Participants (n=12) were largely female (11/12, 92%) and Caucasian (9/12, 75%) with a mean age of 45.8 years (SD 9.6) and a mean BMI of 34.1 kg/m^2^ (SD 3.6). Most (10/12, 83%) were employed full time. Participants' mean BDI-II score was 5.6 (SD 6.2). Most participants reported having more than one social media account (10/12, 83%): 33% (4/12) reported daily log-ins and 42% (5/12) reported greater than daily log-ins.

### Study 3 Results

#### Measures

##### Retention, Attendance, and Engagement

All participants provided weight data at the end of treatment. A total of 2 of the 12 (17%) participants failed to attend the follow-up visit but instead sent their weights via email. Only 6 participants out of 12 (50%) attended the week 6 visit. Participants posted, on average, 130.3 tweets (SD 124.0; median 74.0, IQR 211) over 12 weeks.

##### Weight Loss

Participants lost a mean of 5.4 pounds (SD 6.4; range -14.2 to 3.9) or 3.0% of their baseline weight (SD 3.1). A total of 2 participants out of 12 (17%) provided weight by self-report. Of the 10 out of 12 participants (83%) who had weight measured objectively, mean weight loss was 5.04 pounds (SD 6.20; range -12.8 to 3.9) or 2.4% of their baseline weight (SD 2.9). A total of 42% (5/12) of participants lost clinically significant weight (ie, ≥5% of baseline weight), 33% (4/12) lost less than 5% of their baseline weight, none remained weight neutral, and 25% (3/12) gained weight.

##### Acceptability

Of the 10 participants who completed the acceptability survey, the vast majority of participants found the Twitter group to be at least a somewhat good source of support (9/10, 90%) and information (8/10, 80%) (see Table 4). The majority (7/10, 70%) said they would be at least somewhat likely to continue to use Twitter after the study. Most participants at least somewhat enjoyed all types of posts with 70% (7/10) reporting this for status updates, 80% (8/10) for posing a question, 80% (8/10) for replying to others’ questions, and 90% (9/10) for reading others' posts.

##### Weight-Related Social Support

At follow-up, participants' ratings of frequency and helpfulness of emotional support were not different by relationship category (*P*=.30 to .53). Participants' ratings of frequency of informational support were greater for the Twitter group than for both family (*P*=.01) and friends (*P*=.02), while ratings of helpfulness of informational support were greater for the Twitter group relative to family (*P*=.01), but not friends (*P*=.08). The appraisal support subscales did not differ by relationship category (*P*=.39 to .79). Participants' ratings of positive social influence were significantly different across relationship categories (*P*<.001). Participants rated their Twitter group higher in positive social influence for weight loss than their family (*P*=.03), but not in-person friends (*P*=.21). Ratings of embarrassment (*P*=.11) and how judgmental (*P*=.40) each relationship category was were not significantly different.

#### Focus Group

The focus group was attended by 10 of 12 participants (83%). Five major themes emerged from a total of 35 responses about what participants liked most about the online aspect of the program. The most frequently occurring theme, endorsed by all 10 (100%) participants, was encouragement from the group (eg, “There was always something encouraging on there and nonjudgmental.”). The second-most frequently occurring theme, endorsed by 9 of 10 (90%) participants, was feeling nudged or inspired by others' posts (eg, “Being inspired by other people’s posts, like oh she went for a walk, alright I’ll go too.”). The next two major themes were both mentioned by 6 of 10 participants (60%) and included feeling not alone (eg, “It felt like a secret little society of people on the same path.”) and receiving valuable and relevant information (eg, “I like how you get information like recipes without having to spend time searching the Web.”). Finally, a major theme was instant feedback (eg, “When I went out for a walk and posted it, someone would always see it and favorite it. Instantly.”).

One major theme and three minor themes emerged from a total of 20 responses on what participants disliked most about the online part of the program. The major theme, endorsed by all 10 (100%) participants, was not preferring to follow people other than fellow group members (eg, “Too many tweets in the stream made it hard to find the group tweets at times.”). Minor themes, each endorsed by 2 of 10 (20%) participants, included difficulties navigating the functions of Twitter (eg, “I never used Twitter before, I found it difficult to navigate.”), wanting clearer notification when someone has tweeted them (“I didn’t know how to tell someone was tweeting me.”), no dislikes at all, and privacy concerns (eg, “The thing with Twitter is that anyone can find you.”). While the orientation reviewed navigation, notification functions, and privacy settings, participants were new to Twitter and the amount of information shared in the orientation may have been too much at once. For example, the participant with concerns about being found by anyone on Twitter must not have understood that by using the avatar and pseudonym, along with privacy settings where only users she approves can see her tweets, would all make it impossible for anyone to find her on Twitter. This was clarified when she expressed this concern. The 2 out of 10 (20%) participants who said there was nothing they disliked about Twitter were, not surprisingly, former Twitter users. Finally, 1 participant out of 10 (10%) did not like that the app and online Twitter interfaces differed, 1 (10%) participant did not like to be repeatedly mentioned in tweets when coaches checked in on her, and 1 (10%) mentioned not liking ads in the newsfeed.

#### Lessons Learned From Study 3

Although results were promising in Study 3, some participants still experienced barriers to using the Twitter interface. When using a novel platform, more extensive training in the platform is likely needed. Our orientation lasted 2 hours and was held in a group format which may not have met the needs of individuals who needed more intensive help. Individual orientation meetings and a prestudy trial period to make sure users achieve comfort with the modality might prevent usability issues. Another approach is to recruit users of the platform being used in the study. This would circumvent the learning curve of the social media platform. Previous studies have used both Twitter [30] and Facebook [31] in weight-loss interventions with success. Another lesson learned is that participants seemed more interested in hearing from each other than in following relevant sources or blogs. In our future work we will use our feed to push select resources and information to users rather than recommend they follow relevant feeds. This will also allow us to better moderate what content they receive, which may be important given how plentiful misinformation is on social media. Who a participant follows seems best determined by the participant, with some preferring very small networks, while others prefer growing their networks. We also learned that participants enjoy each other’s posts, but at the same time many participants experienced anxiety about posting about themselves. In our future work we will examine whether incentivizing some participants to post regularly will increase engagement of others via role modeling processes, but also enhance the group’s experience since they enjoy hearing from each other. Future studies should explore novel ways to induce meaningful engagement that involves sharing of experiences.

## Results

Table 1 shows the sample characteristics from the three iterative pilot studies. Table 2 shows the sample characteristics, weight loss, and social media engagement for each of the three interventions. Table 3 shows acceptability of the Twitter social network by participants. Table 4 shows the weight-related social support of participants by relationship category at 12 weeks.

**Table 1:** Sample characteristics of three iterative pilot studies.

Characteristics	Study 1 (n=10)	Study 2 (n=11)	Study 3 (n=12)
Age (years), mean (SD)	46.2 (10.9)	48.4 (12.3)	45.8 (9. 7)
Baseline BMI^a^ (kg/m^2^), mean (SD)	35.9 (4.6)	33.8 (3.7)	34.2 (3.6)
Female, n (%)	9 (90)	11 (100)	11 (92)
Caucasian, n (%)	8 (80)	9 (82)	9 (75)
Employed full time, n (%)	8 (80)	10 (91)	10 (83)
Ever used social media, n (%)	8 (80)	11 (100)	12 (100)
Has Facebook account, n (%)	6 (60)	11 (100)	12 (100)
Ever had Twitter account, n (%)	1 (10)	5 (45)	1 (8)
Have ever used online community for weight loss, n (%)	2 (20)	6 (55)	2 (17)

^a^Body mass index (BMI).

**Table 2:** Intervention and sample characteristics, weight loss, and engagement

Characteristics	Study 1 (n=10)	Study 2 (n=11)	Study 3 (n=12)
Intervention	12 group visits + Twitter	12 group visits + Twitter	1 group visit + Twitter
Length	12 weeks	12 weeks	12 weeks
Depression status	Depressed	Nondepressed	Nondepressed
Social media inclusion criteria	None required, not an active Twitter user	Daily Facebook use required, not an active Twitter user	Daily Facebook use required, not an active Twitter user
Technology inclusion criteria	Internet access at home	Internet access at home	Internet access at home, has a mobile phone
Weight change (lbs), mean (SD)	-2.3 (7.7)	-5.6 (6.3)	-5.4 (6.4)
Weight change (%), mean (SD)	1.2 (3.6)	3.0 (3.4)	3.0 (3.1)
≥5% weight loss, n (%)	2 (20)	4 (36)	5 (42)
Tweets, mean (SD)	110.8 (112.4)	121.9 (127.1)	130.3 (124.1)

**Table 3:** Acceptability of Twitter social network.

Questions from acceptability survey		Study 1 (n=9)	Study 2 (n=9)	Study 3 (n=10)
**To what extent did you find Twitter to be useful as a source of support for your weight-loss effort?**				
	Score, mean (SD)	3.0 (4.2)	8.0 (2.0)	6.8 (1.9)
	Rating a 5 or greater^a^, n (%)	6 (67)	9 (100)	9 (90)
**To what extent did you find Twitter to be useful as a source of information about weight loss?**				
	Score, mean (SD)	5.5 (6.4)	8.2 (2.6)	7.2 (2.3)
	Rated a 5 or greater, n (%)	6 (67)	8 (89)	8 (80)
**How likely is it that you will continue to use Twitter after the study has ended?**				
	Score, mean (SD)	5.1 (4.1)	8.1 (2.4)	5.8 (3.2)
	Rated a 5 or greater, n (%)	6 (67)	9 (100)	7 (70)
**How much did you like posting a status update about yourself?**				
	Score, mean (SD)	4.9 (3.9)	6.0 (2.6)	6.6 (2.4)
	Rated a 5 or greater, n (%)	5 (56)	7 (78)	7 (70)
**How much did you like posting a question?**				
	Score, mean (SD)	4.7 (4.0)	6.9 (3.0)	6.3 (2.9)
	Rated a 5 or greater, n (%)	5 (56)	8 (89)	8 (80)
**How much did you like replying to a question posed by someone else?**				
	Score, mean (SD)	5.1 (4.2)	7.4 (1.9)	6.8 (3.1)
	Rated a 5 or greater, n (%)	6 (67)	9 (100)	9 (90)
How much did you like reading others' posts?				
	Score, mean (SD)	5.3 (4.0)	8.9 (1.8)	8.1 (2.4)
	Rated a 5 or greater, n (%)	5 (56)	9 (100)	9 (90)

^a^All response options were rated on a scale from 0 to 10 from *not at all (likely)* to *very much/likely*.

**Table 4:** Weight-related social support by relationship category at 12 weeks.

Social support		Study 1, mean (SD)	Study 2, mean (SD)	Study 3, mean (SD)
**Emotional support: frequency**				
	Twitter group	2.3 (0.9)	2.8 (0.5)^a^	2.6 (1.1)
	Family	2.3 (0.7)	2.0 (1.0)	2.3 (1.2)
	Friends	1.9 (0.7)	1.7 (0.6)	2.2 (1.0)
**Emotional support: helpfulness**				
	Twitter group	2.7 (1.2)	3.3 (1.0)^b^	3.1 (1.2)
	Family	2.5 (0.8)	2.4 (1.5)	2.6 (1.2)
	Friends	1.9 (0.7)	2.1 (1.2)	2.8 (1.3)
**Informational support: frequency**				
	Twitter group	2.7 (1.1)^a^	3.2 (0.7)^a^	3.1 (1.2)^a^
	Family	1.5 (0.5)	1.7 (0.8)	1.6 (0.5)
	Friends	1.8 (0.7)	1.7 (0.6)	1.8 (0.9)
**Informational support: helpfulness**				
	Twitter group	3.1 (1.5)^a^	3.6 (1.1)^a^	3.5 (1.3)^c^
	Family	1.5 (0.4)	2.1 (1.1)	1.9 (0.8)
	Friends	1.8 (0.7)	2.2 (1.2)	2.4 (1.2)
**Appraisal support: frequency**				
	Twitter group	1.8 (0.5)	2.2 (0.8)	2.3 (1.3)
	Family	1.8 (0.7)	2.1 (1.0)	2.1 (0.8)
	Friends	1.8 (0.8)	2.1 (1.1)	2.0 (0.8)
**Appraisal support: helpfulness**				
	Twitter group	1.9 (0.7)	2.8 (1.2)	2.8 (1.6)
	Family	2.1 (0.8)	2.5 (1.5)	2.8 (1.3)
	Friends	2.2 (1.4)	2.7 (1.7)	3.2 (1.2)
**Positive composite score**				
	Twitter group	19.2 (7.8)	21.6 (1.7)^a^	19.7 (3.4)^c^
	Family	14.8 (4.5)	14.4 (5.7)	15.7 (5.7)
	Friends	15.7 (5.3)	17.4 (4.9)	18.3 (4.6)
**Embarrassment**				
	Twitter group	1.5 (0.8)	2.6 (1.7)	1.9 (1.2)
	Family	3.0 (1.9)	3.9 (1.3)	3.3 (1.4)
	Friends	3.8 (1.6)	3.6 (1.7)	2.8 (1.3)
**Judgmental**				
	Twitter group	1.2 (0.4)	1.4 (0.7)^a^	1.8 (1.3)
	Family	3.2 (1.5)	3.3 (1.4)	2.7 (1.6)
	Friends	2.2 (1.6)	2.3 (1.3)	2.2 (1.3)

^a^ *P*<.05 for family and friends.

^b^ *P*<.05 for friends only.

^c^ *P*<.05 for family only.

In summary, retention rates were high, ranging from 81 to 100% across the pilots. Participants tweeted, on average, 9.3 to 10.8 tweets per week. Although we did not track the distribution of the tweets across time, the rate of engagement was more than one tweet per day over the 12 weeks. In terms of weight loss, participants with depression had the lowest rate (20%) of clinically significant weight loss (≥5% of baseline weight), while 36 to 42% of nondepressed participants lost clinically significant weight. The pilot that included only one intervention visit (Study 3) did not reveal a trend toward less weight loss compared to the studies that had weekly intervention visits (42% vs 20 to 36% losing clinically significant weight), which supports moving to the next step of a fully powered randomized trial to compare Twitter-delivered versus traditionally delivered intervention on weight loss. Across all three studies, participants rated their Twitter group as at least as good as, or a significantly greater source of, weight-related social support than their close ties (ie, family and friends). Focus groups for all three studies revealed similar major themes about what was liked most about the online social network, including social support, encouragement, and nudging. The major themes about what was liked the least about the online social network were also similar across studies, including feeling overwhelmed by too many feeds to follow and the usability of Twitter.

## Discussion

### Principal Findings

This series of studies showed that using a private Twitter group as an adjunct to an in-person behavioral weight-loss intervention is feasible and acceptable in a sample of adults with obesity who did not have depression and who were regular users of social media (though Twitter-naïve). Among adults with depression who had less social media experience, this approach did not appear to be very acceptable. Although providing participants access to each other and counselors via online social networks conceivably could provide greater opportunities for social support, participants appear to need a certain comfort level with this modality to actively engage. In each study, we provided participants with an orientation visit to teach them to use the online social network; however, previous experience using an online social network may be more instrumental to their engagement and success than study-provided training. In Study 1, participants with depression reported that they were not sure how to solicit social support in the online setting. Greater guidance may be needed to activate patients to solicit social support via this modality. For example, giving patients specific ideas on what to post, putting them in communities with highly active and engaging users that may serve as role models, and proactively drawing them into conversations to ignite engagement could be tested in future work.

Among nondepressed adults attempting to lose weight, using an online social network as either an adjunct or as the primary intervention modality appears to be both acceptable and feasible. Retention was 82% when Twitter was used adjunctively (Study 2) and 100% when Twitter was used nearly exclusively as the intervention modality (Study 3). Thus, we found no reason to believe that offloading content to an online social network negatively impacts retention. In fact, the online social network modality may be particularly conducive to retention given that the usual barriers to participation in visit-based programs (eg, weather, schedule constraints, and travel) are not barriers in an online social network-based program. The single intervention visit was not likely deemed important or necessary to participants as it was difficult to schedule and only attended by 50%, so did not likely add much value. Mean weight loss observed across the pilots that recruited nondepressed adults (5.5 lbs in 12 weeks or 0.5 lbs per week) was fairly comparable to that achieved in the Diabetes Prevention Program Lifestyle Intervention (ie, mean 14.33 lbs in 24 weeks or 0.58 lbs per week), especially considering the latter had a far higher percentage of males than the samples of our pilot studies (32% vs 4.3%), and men achieved greater weight loss than women [32]. A randomized controlled noninferiority trial evaluating whether a Twitter-delivered behavioral weight-loss intervention is not appreciably worse than the traditionally delivered version is a next needed step to explore the efficacy of this approach when conducted over a full year, the usual length of behavioral weight-loss interventions. Whether participants will continue to engage in a longer-term lifestyle intervention administered entirely via an online social network is unknown.

Future research should explore whether adding a social network to a traditional behavioral weight-loss intervention improves weight-loss outcomes relative to a traditional version that does not include a social network. It remains unclear if the social network adds value or if it just adds more burden to an already burdensome intervention. Studies could explore the influence of the social network on social support and on attendance to group visits. One possible unexpected outcome could be reduced attendance at group visits to the extent that participants feel they can get enough information and support from the social network alone. Our study could not address this question directly, but we did observe poor attendance at the single study visit offered in the third pilot.

Findings extend previous research that showed that people using Twitter to talk about their weight loss rated their Twitter connections as more supportive and less judgmental than family and friends [28]. In Studies 2 and 3, we found that that the weight-related support participants experienced from their Twitter group exceeded that which they experienced from family and/or friends. The accessibility of an online group may increase opportunities for individuals to receive social support and the shared goals may strengthen the support received, even if these are loose social ties. Future studies using online social networks should examine ways to further enhance users' experience of social support, as this would be particularly instrumental for individuals who receive insufficient support for their weight-loss efforts elsewhere in their lives [5,28].

Behavioral interventions delivered via online social networks require the translation of intervention materials into a format that resembles communication habits of the social network. We created a content library that included online articles, brief headlines to be used in tweets to entice participants to click on and read the articles, exercise videos on YouTube, links to recipes from reputable sources, and other online resources. We developed orientation materials to advise participants on what to expect and how to interact when using this modality, and trained counselors in “microcounseling,” a way to deliver counseling in a brief, asynchronous manner each day as opposed to in discrete chunks as is characterized by 60- to 90-minute visits that occur once a week.

Emerging research is shedding light on the type of content of posts most likely to be viewed, clicked, and read in weight-loss social networks. One study showed that polls, suggestions, and posts querying participants' weight-loss progress received the highest levels of participant engagement in a Facebook weight-loss group [8], and another found that polls and photos received the highest levels of engagement in a Facebook weight-loss group [33]. Research is needed to explore the timing of posts most likely to lead to clicks on links and the length of articles and videos that are most likely be read and viewed. To be successful in changing behavior, the online social network modality will require great attention to these issues. In our iterative series of pilot studies, where Study 1 informed Study 2, which then informed Study 3, we gathered qualitative and experiential data, and we continue to iterate these features in subsequent studies to identify best practices using online social networks as a behavioral intervention modality.

Future work should explore the best means for leveraging online social networks for weight loss in patients with depression. Barriers to engagement should be identified. Although none of our participants discussed worsening depressive symptoms or suicidality in the online social network, procedures for handling such crises should be established in advance. Participants were advised to call us if they had a crisis to report as opposed to posting it online or in a private message. Counselors logged in twice a day, 7 days a week, to closely monitor participants’ posts. We recommend that counselors have a frequent and regular presence in social network-delivered interventions, not only to monitor posts but to stimulate engagement.

### Limitations

This study has limitations. Our samples were predominantly female and Caucasian. A review of male representation in weight-loss trials revealed that, on average, only 27% of samples across trials are male [34]. Our study showed even lower rates of males with 2 males across all 33 participants (6%). It is notable that our male participants were among the lowest engagers, with one tweeting twice (he had no previous experience with social networking, no mobile phone, and reported only using his computer for email) and the other only seven times (he reported that he was hoping for more competition-style programming and would have preferred Facebook). Research is needed to determine how best to design social media-delivered programs for men, as this type of program may not be the best fit.

Because of challenges in utilizing the Twitter modality, we only included participants in Studies 2 and 3 who were current users of any other online social network, which limits generalizability. However, research from the Pew Internet & American Life Project found that 74% of online Americans have at least one social media account [23]. In all studies, most individuals had no experience with Twitter, which may have affected their comfort level in engaging with and using this platform. Focus group data revealed that problems understanding the Twitter interface was a barrier for some participants. Future studies might find better results by recruiting users of the same online social network used to deliver the intervention. By doing so, participants would not have to initiate a new social network habit (ie, checking another feed), time would not have to be allocated to training on the social network, and intervention content would appear in their usual feed. A potential challenge of using commercial online social networks is the lack of control over the user interface and unexpected changes in settings and features. We experienced no problematic changes in settings and features in our pilot series, but these could arise in future interventions and treatment providers must remain vigilant of these changes so they do not impact participant confidentiality. The challenges of using investigator-developed platforms include lack of experienced users, loss of opportunity for content to appear in participants' regular social media feeds, and development that will require a great deal of time and money.

### Conclusions

Using commercial online social network platforms like Twitter to deliver behavioral weight-loss counseling may be a less expensive and more convenient alternative to traditional modalities that require numerous clinic visits. Findings have broad implications for behavioral interventions as this modality could be used for a variety of interventions provided that materials are appropriately translated for delivery in an online social network modality. Future research is needed to refine the populations for whom this modality is ideal, to test the efficacy of interventions delivered entirely via online social networks, and to design and deliver content in the most engaging and effective ways using this modality.

## Abbreviations

BDI-II: Beck Depression Inventory II
BMI: body mass index
DPP: Diabetes Prevention Program
IQR: interquartile range
NIH: National Institutes of Health
UMass: University of Massachusetts
WMSI: Weight Management Support Inventory
